# Hydraulic constraints determine the distribution of heteromorphic leaves along plant vertical height

**DOI:** 10.3389/fpls.2022.941764

**Published:** 2022-09-29

**Authors:** Xiao-Dong Yang, Elhamjan Anwar, Yi-Lu Xu, Jie Zhou, Long-Bin Sha, Xue-Wei Gong, Arshad Ali, Yong-Chao Gao, Yanju Liu, Ping Ge

**Affiliations:** ^1^ Department of Geography and Spatial Information/Center for Land and Marine Spatial Utilization and Governance Research, Ningbo University, Ningbo, China; ^2^ Institute of Resources and Environment Science, Xinjiang University, Urumqi, China; ^3^ Global Centre for Environmental Remediation (GCER), The University of Newcastle (UON), Newcastle, NSW, Australia; ^4^ Institute of Applied Ecology, Chinese Academy of Sciences, Shenyang, China; ^5^ Forest Ecology Research Group, College of Life Sciences, Hebei University, Baoding, Hebei, China; ^6^ Shandong Provincial Key Laboratory of Applied Microbiology, Ecology Institute, Qilu University of Technology (Shandong Academy of Sciences), Jinan, China; ^7^ Department of Development Planning, Zhejiang Gongshang University, Hangzhou, China

**Keywords:** anatomical structures, arid desert region, cellular water relations, drought stress, morphological traits, water transport capacity

## Abstract

As an interesting and important trait of some drought-tolerant species, heteromorphic leaves are distributed differentially along plant vertical heights. However, the underpinning mechanism for the formation of heteromorphic leaves remains unclear. We hypothesize that heteromorphic leaves are caused by the hydraulic constraints possibly due to the compensation of the changes in functional traits in response to water transport capacity or the reduction of ineffective water loss. In this study, differences in water transport capacity, morphological traits, anatomical structures, and cellular water relations among three typical types of heteromorphic leaves (i.e., lanceolate, ovate, and broad-ovate) of *Populus euphratica* Oliv. (a dominant species of desert riparian forest in Central and West Asia) and their relationships were analyzed in order to explore the forming mechanism of heteromorphic leaves. The results showed that the lanceolate, ovate, and broad-ovate leaves were growing in the lower, intermediate, and higher positions from the ground, respectively. Morphological traits, anatomical structures, cellular water relations, and water transport capacity significantly varied among the three types of heteromorphic leaves (*P*< 0.01). Drought stress in broad-ovate leaves was significantly higher than that in ovate and lanceolate leaves (*P*< 0.01). Water transport capacity has significant correlations with morphological traits, anatomical structures, and cellular water relations (*R* ^2^ ≥ 0.30; *P*< 0.01). Our results indicated that heteromorphic leaves were used as an important adaptive strategy for *P. euphratica* to alleviate the increase of hydraulic constraints along vertical heights.

## Introduction

Leaves are the most important organs for determining overall plant growth because they carry out many physiological activities such as photosynthesis, transpiration, respiration, and photoreception (Coble et al., 2017; Wang and Jiao, 2020). Compared with other plant components, leaves have attracted much attention due to their observability and susceptibility to environmental changes (Wright et al., 2004). Revealing leaf–environment relationship has been regarded as one of the core problems in plant physiology and functional ecology (Poorter et al., 2010a; Ke et al., 2022). At present, although many scholars have carried out a large number of studies about leaf ecological response from biochemical stoichiometry, twig–leaf tradeoff relationship, economic spectrum, and functional traits to environmental stress (Chartzoulakis et al., 2002; Sessa and Givnish, 2014; Yang et al., 2014a; Pan et al., 2016), changes of the leaf morphology with environmental gradient, especially at a small scale, such as along the vertical height in a tree, are still relatively insufficient (Iqbal et al., 2017; Zhao et al., 2021).

Heteromorphic leaves are an important and interesting manifestation of leaf morphology, which refers to the fact that plants with the same genotype grow different shaped leaves (e.g., lanceolate, ovate, and broad-ovate) along vertical heights (Hao et al., 2017; Iqbal et al., 2017). In nature, heteromorphic leaves are commonly found in aquatic plants in the transition zones between land and water (Li et al., 2017). It is believed that heteromorphic leaves reflect the plants’ adaptation to environmental changes *via* their phenotypic plasticity (Zhao et al., 2021). The study of their change and formation mechanism can reveal the multiple effects of the environment on plants from gene to population (Li et al., 2017). Over the past multidecade, many studies have explored the functional and structural differences (i.e., anatomical structures, morphological and physiological traits) among different types of heteromorphic leaves and their responses to environmental changes (Niinemets et al., 2004; Liu et al., 2015; Xu et al., 2016). However, the understanding regarding the underlying mechanisms for the formation of heteromorphic leaves remains unclear.

Hydraulic constraints is likely to be one of the major mechanisms for explaining the distribution of heteromorphic leaves along plant vertical heights (Leigh et al., 2011). As plants grow taller, the increases of the gravitational potential between soil and the top of the canopy and the frictional resistance of water flow in xylem would enlarge the leaf-to-atmosphere vapor pressure deficit (leaf-to-air VPD) and thus increase xylem tension (Koch et al., 2004; Ryan et al., 2006; Yang et al., 2019a) . The progressive drop in xylem pressure can trigger embolism, which then result in plant hydraulic dysfunction (Ryan and Yoder, 1997; Mcdowell et al., 2010; Yang et al., 2022b). To reduce the risk of xylem embolism caused by increased height, plants usually improve their water transport capacity while reducing ineffective water loss to alleviate hydraulic constraints *via* the adjustment of water-related functional traits (Diaconu et al., 2016; Garcia et al., 2021). Compared with other traits, adjustment of leaf morphology has been proven to be more related to hydraulic efficiency and reducing transpiration water loss because of its higher plasticity and as a water exchanger between plants and atmosphere (Sassi et al., 2013; Tian et al., 2016; Males, 2017). As an interesting and special form of leaf morphological change, variation of heteromorphic leaves with vertical height may be used by plants to alleviate hydraulic constraints.

**Figure 1:**
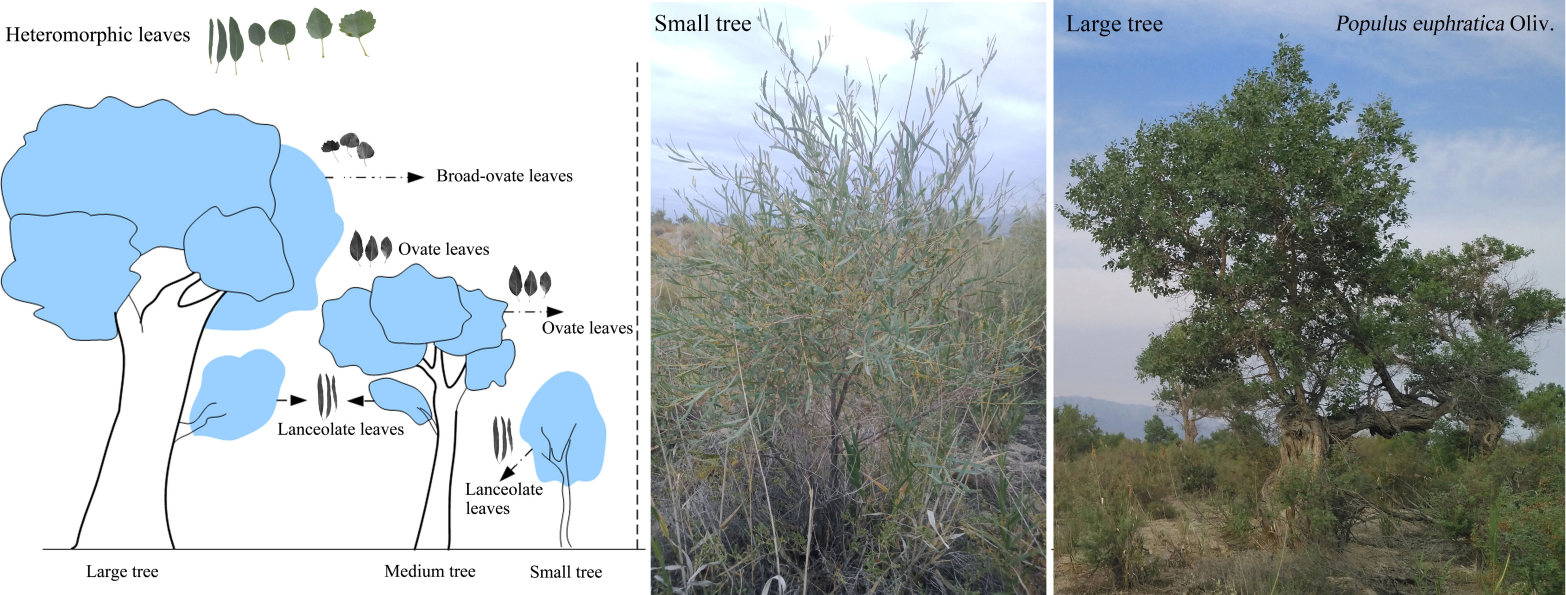
Spatial distribution of the three types of heteromorphic leaves.

*Populus euphratica* Oliv., a dominant species of desert riparian forest in Central and West Asia (Yang et al., 2014b), is one of the few tree species in the terrestrial ecosystems that develops heteromorphic leaves along vertical heights (**Figure 1**) (Li and Zheng, 2005; Liu et al., 2015; Zhai et al., 2020). Previous studies have confirmed that the annual precipitation in the habitat of *P. euphratica* is generally no more than 200 mm, while annual potential evaporation exceeds 1,500 mm (Hao et al., 2017; Yang et al., 2019b; Shi et al., 2021), which made it subject to long-term drought stress and an increase of the leaf-to-air VPD with vertical heights (Keram et al., 2019; Li et al., 2019). It is known that drought stress increases the risk of xylem embolism, making plants from water-limited environments more prone to hydraulic dysfunction relative to those inhabiting water abundance-rich environments (Christian, 2019; Garcia et al., 2021). In order to reduce the extra hydraulic constraints caused by drought stress, adjustment of leaf morphology of *P. euphratica* may be more drastic than that of other plants (Zhuang et al., 2011; Liu et al., 2015; Yang et al., 2022b). A dramatic result is an obvious change in leaf shape (Li et al., 2019; Zhai et al., 2020). Different types of heteromorphic leaves would succeed at different vertical heights (**Figure 1**). At present, many studies have found the obvious differences in morphological traits and their associated anatomical structures and cellular water relations among different types of heteromorphic leaves (Yang et al., 2005; Xu et al., 2016; Iqbal et al., 2017); however, evidence revealing the linkage between the formation of heteromorphic leaves and hydraulic constraints is still scarce (Li et al., 2019). In this study, we aim to explore whether hydraulic constraints affect the distribution in different types of heteromorphic leaves along *P. euphratica*’s vertical heights. We hypothesize that heteromorphic leaves are caused by the hydraulic constraints possibly due to the compensation of the changes in morphological traits and their loaded anatomical structures and cellular water relations to water transport capacity or water loss along vertical heights.

## Materials and methods

### Experimental site

The study site is located in the Ebinur Lake Wetland Nature Reserve (82°36′–83°50′E, 44°30′–45°09′N) in the southern part of the Gurbantunggut Desert, Xinjiang Uygur Autonomous Region, in western China. The site belongs to a temperate continental climate with an annual sunshine hour of approximately 2,800 h, while mean annual precipitation is less than 100 mm, and annual potential evaporation ranges from 1,500 to 2,000 mm. The annual temperature ranges from 44°C to -33°C, with an average temperature of all years ranging from 6°C to 8°C. Mean temperature in the warmest month (July) is 27°C, while that in the coldest month (January) is about -17°C (Wang et al., 2021). Affected by sparse rainfall and the extremely arid climate, the zonal plant community is mainly composed of desert plants. The most common plant community types are desert riparian forest and sparse xerophytic shrubs (Zhang et al., 2018). *Haloxylon ammodendron* (C. A. Mey.) Bunge and *P. euphratica* are the dominant species of these two common communities, respectively (Gong et al., 2019). *P. euphratica* is the only tree species in the local plant community; the maxima of its crown area and height are close to 20 m^2^ and 8 m, respectively (Yang et al., 2014b).

### Experimental plot and sample collection

This study was carried out in a 100 m × 100 m long-term dynamic observation and research field of *P. euphratica* community established by the Xinjiang University. The field had a moderate slope and sandy soil, where 76 P*. euphratica* individuals grow, and vegetation coverage was approximately 30%.

*P. euphratica* is one of the deciduous arborescent species with numerous and simultaneous types of leaf shapes, i.e., linear, lanceolate, ovate, dentate ovate, dentate rhombic, dentate broad-ovate, and dentate fan-shaped leaves (**Figure 1**) (Liu et al., 2015). In this study, all heteromorphic leaves were categorized into three typical types based on morphological properties: lanceolate, ovate, and broad-ovate leaves, as suggested by Hao et al. (2017). It is known that leaf shapes of *P. euphratica* change with vertical heights (Iqbal et al., 2017; Zhai et al., 2020). As suggested from Long et al. (2021), *P. euphratica* individuals can be divided into small (height<2.0 m), medium (2.0 ≤ height ≤ 4.5 m), and large (height >4.5 m) trees by using its height. The lanceolate leaves grow near the ground (i.e., all crown of the small trees and the bottom of the medium and large tree crowns), while the ovate leaves distributed in the higher vertical position (i.e., the top of the medium tree crowns and the intermediate of the large tree crowns), but the broad-ovate leaves only located at the top of the large tree crowns (**Figure 1**).

In this study, nine *P. euphratica* individuals (three individuals for small, medium, and large trees, respectively) were haphazardly selected as sampling objects in the long-term dynamic observation and research field. Three experimental groups were categorized according to the growth positions of the three typical types of heteromorphic leaves for the nine individuals: 1) nine sampling regions of lanceolate leaves, 2) six sampling regions of ovate leaves, and 3) three sampling regions of broad-ovate leaves. The top of the large tree crown was divided into two sampling regions according to the east–west direction in order to reduce the impact of unbalanced sample size on the statistical analysis results due to fewer sampling regions of broad-ovate leaves than that of the other two leaves. Thus, lanceolate, ovate, and broad-ovate leaves have nine, six, and six sampling regions, respectively (**Table 1**).

**Table 1:** Differences in vertical distributions and sampling regions among the three types of heteromorphic leaves.

Plant size	Individual number	Height (m)	Crown area (m^2^)	Heteromorphic leaves		Spatial positions of heteromorphic leaves in a crown	Sampling regions (m)
Small tree	3	2.16±0.05	0.84±0.16		Lanceolate	All	<2.0
Medium tree	3	3.73±0.31	3.30±0.59		Lanceolate	Bottom	<1.5
					Ovate	Intermediate and top	1.5 ≤ height ≤ 4.5
Large tree	3	7.16±0.35	20.77±7.63		Lanceolate	Bottom	<1.5
					Ovate	Intermediate	1.5 ≤ height ≤ 3.5
					Broad-ovate	Top	>3.5

### Measurements of morphological traits, anatomical structures, and cellular water relations

In this study, we used two aspects to measure the response of leaf shape on the vertical height (**Table 2**). The first was the morphological traits, which described differences in morphology and appearance among heteromorphic leaves. The other was the internal anatomical structures and cellular water relations of leaves, which were both closely related to leaf morphological change, because they are the structural and physiological determinants of the leaf appearance (Li and Zheng, 2005; Song et al., 2021). Morphological traits included the area; thickness, length, and width of leaf; and petiole length and diameter. Anatomical structures were composed of the thickness of the epidermis, cuticle, palisade, and outer cell wall. Cellular water relations included the apoplastic water fraction, osmotic potential at full turgor, and average modulus of elasticity.

**Table 2:** The main traits used to test the hypothesis in this study.

Classification	Subclassification	Trait names
Morphological traits and their loaded anatomical structures and cellular water relations	Morphological traits	Leaf area (cm^2^) and thickness (μm)
		Leaf length (cm) and width (cm)
		Petiole length (cm) and diameter (cm)
	Anatomical structures	Epidermis thickness (μm)
		Cuticle thickness(μm)
		Palisade thickness (μm)
		Outer cell wall thickness (μm)
	Cellular water relations	Apoplastic water fraction (%)
		Osmotic potential at full turgor (MPa)
		Average modulus of elasticity (Mpa)
Water transport capacity	Stoma density (n·mm^-2^) and size (μm)	
	Vessel density (n·mm^-2^) and diameter (μm)	
	Huber value	
	The instantaneous water conductivity (*K_wb_*) of branches (kg·s^-1^·m^-2^·Mpa^-1^)	
	Leaf specific hydraulic conductance (*K_l_*) (kg·s^-1^·m^-2^·Mpa^-1^)	
	Transpiration traits [transpiration rate and stomatal conductance (Tr and Cond)] (H_2_Ommol·m^-2^·s^-1^)	
Drought stress on plants	Midday leaf water potential (MPa)	
	Malondialdehyde (MDA) content (μg·g^-1^)	
	Proline content (μg·g^-1^)	
	Soluble sugar content (μg·g^-1^)	

For the measurement of the morphological traits and their loaded anatomical structures and cellular water relations, a high branch scissors was used to cut one big peripheral branch from each sampling region haphazardly from local time 6:00 a.m. to 7:00 a.m. in July 2018 and immediately placed those branches into a bucket filled with water, which were then brought back to the field experimental station of the Xinjiang University. The chopped branch had a diameter >3 cm and at least included two branch levels. Zero level was twig, while the first level was the primary branch that is in front of the twig (Yan et al., 2013). Three to five healthy and undamaged leaves were haphazardly picked from each chopped branch to determine leaf morphological traits. Specifically, leaf area was measured with a leaf area meter (LI-3100C, LI-COR, USA). The thickness, length, and width of the leaf and length and diameter of petiole were measured with a Vernier caliper.

The anatomical traits were measured using microscopic observation and paraffin section method. Three to five healthy and undamaged leaves were haphazardly chosen from each chopped branch. The paraffin slides were prepared according to the method of Luo et al. (2012). Specifically, small pieces (1 cm^2^) were haphazardly taken from each leaf and then were fixed by a mixture of 5% formaldehyde, 5% glacial acetic acid, and ethanol, followed by dehydration in a graded ethanol series. Fixed samples were embedded in the paraffin and sectioned to 5-μm size using a rotary microtome (RM 2235, Leica, Germany). Slides were stained in an aqueous 0.1% toluidine blue O solution for 1–2 min and then dehydrated with anhydrous alcohol. After that, the slides were placed in the oven to dry, made transparent with xylene, and sealed with neutral resin. The slides were measured on an optical microscope (CX33, Olympus, Tokyo, Japan). The measurement units of all observation indices were “μm.” Each index was measured at least 30 times, and the final value was expressed as the mean of those views. Statistically, when the data show normal distribution, 30 was considered as the minimum sample size to eliminate statistical bias.

Cellular water relations were obtained from a pressure-volume (P-V) isotherm. Three to five healthy and undamaged leaves were randomly chosen from each sampling region from local time 6:00 a.m. to 7:00 a.m. and then enclosed in a plastic bag prior to being transported to the field experimental station. The P-V isotherms were measured using a pressure bomb (portable plant moisture system SKPM 1400, Skye Instrument Ltd., UK). Kl and Huber values.

### Measurements of water transport capacity

In this study, stoma density and size, stem xylem anatomy (vessel diameter and density), Huber value, instantaneous water conductivity (*K_wb_*) of branches, leaf specific hydraulic conductance (*K_l_*), and transpiration traits [transpiration rate and stomatal conductance (Tr and Cond)] were used as proxies for water transport capacity. These indicators were chosen as proxies because of two aspects: the power source of water transport or its direct influence on the water transport process from stem to leaf (Poorter et al., 2010b; Yang et al., 2022b) (**Table 2**).

Stoma density and size were measured using microscopic observation and paraffin section method in sync with the other anatomical traits (see introduction in the previous section). In terms of the measurement of stem xylem anatomy, a short segment (about 4 cm) was cut from the middle of a long branch with leaves in each sampling region. The segments were immersed in FAA solution. After hardening, about 10–20-μm-thick discs were cut with a sliding rotary microtome (RM 2235, Leica, Germany), mounted on microscope plates, and submersed in the hematoxylin–eosin staining solution. The samples were then dehydrated, oven-dried, and sealed in a similar manner to leaf anatomical samples (Poorter et al., 2010b; Song et al., 2021). After that, the cross sections of two to three samples were photographed using a digital camera (DP73, Olympus, Tokyo, Japan) connected with a light microscope (BX53F, Olympus, Tokyo, Japan) for each sampled segment. Images were analyzed with the software ImageJ *via* particle analysis-function to estimate the diameter and density of the vessels by means of lumen area. At least 30 images were taken for each sample. Vessel diameter and density were averaged for all image fields of each segment.

Cond and Tr were measured using a portable photosynthetic apparatus (Li-6400XT, LI-COR, Lincoln, NE, USA). An artificial leaf chamber and LI-6400-2B red-blue light source were used in order to reduce the influence of the microenvironment on measured results. Light intensity, temperature, and CO_2_ concentration were set at 1,600 μmol·m^-2^·s^-1^, 35°C, and 400 μmol·mol^-1^, respectively, as suggested by Zhao et al. (2021). The measurements were conducted from 9:00 a.m. to 11:00 a.m. local time. An *in vitro* measurement method illustrated by Meng et al. (2019) was adopted, as leaves in the middle and top of large tree crowns were too high to be measured *in situ*. The Cond and Tr were the maximum values of the three types of heteromorphic leaves in our experimental sites as the local maximum light intensity, optimum temperature, and CO_2_ concentration for plant growth in vigorous growing period (mid-July) were used in the measurements. It is worth noting that such treatment eliminated the influences of seasonal and microenvironmental changes in photosynthetic traits on our research purposes (Yang et al., 2022b).

One to three branches with leaves (length ≥25 cm) were haphazardly cut out in each sampling region before dawn (local 6:00 a.m.–7:00 a.m.) in July 2021. As suggested from Gong et al. (2021) and Yang et al., 2022b , the *K_wb_* of branches was measured using a high-pressure flow meter (HPFM-GEN3, Dynamax Inc., Houston, USA) at the field experimental station. The diameter (*D*, m) was measured with Vernier calipers. The total area of all leaves on branch was measured using a leaf area meter (LI-3100C, LI-COR, USA). Equations 1–3 were used to calculate the cross-sectional area of branch (*CSA*; m^2^), *K_l_* and Huber values.


(1) $$K_{l}=K_{wb}/Total\;area\;of\;all\;leaves\;on\;branch$$

(2) $$CSA=\pi \times {\left(D/2\right)}^{2}$$

(3) Huber values=CSA/Total area of all leaves on branch

### Measurements of drought stress on leaves

In order to test whether drought stress aggravates the impact of hydraulic constraints on water transport in *P. euphratica*, we measured the variances of four common physiological proxies of drought stress (midday leaf water potential and the contents of MDA, proline, and soluble sugar) among the three heteromorphic leaves. Here MDA, proline, and soluble sugar can be used as the proxies because they are important osmotic regulatory substances and their contents are closely related to plant drought resistance (Wang et al., 2017). Midday leaf water potential was chosen because it can assess the degree of water shortage in plant (Gong et al., 2021). It is worth noting that these three proxies may be useful for water transport in plants, but they have no direct effect and therefore are not classified as an indicator reflecting water transport. According to the method of Yang et al. (2022b), the leaves of nine sampling individuals were collected from local 12:00 p.m. to 2:00 p.m. in July 2021. All samples were placed in ziplock bags, stored in a 0°C to 4°C refrigerator, and brought back to the laboratory. Then, the samples were first removed from the refrigerator, and the midday leaf water potential was measured with a Water Potential Meter (WP4-C, Degacon Devices Inc., Pullman, WA, USA). After that, as suggested by the methods of Li (2000), the refrigerated fresh leaves were taken out again and cut into small pieces. The contents of MDA, proline, and soluble sugar were measured by thiobarbituric acid colorimetry, sulfosalicylic acid colorimetry, and anthrone colorimetry, respectively. The measurements were repeated at least three times for each experimental sample.

### Statistical analyses

One-way ANOVA was performed to examine the differences in morphological traits, anatomical structures, cellular water relations, water transport capacity, and drought stress among the three types of heteromorphic leaves. The least-square mean separation with Duncan’s correction was used to test the paired differences if the variance of the above traits was homogeneous. Alternatively, Tamhane’s T3 test was used. Principal component analysis (PCA) was used for dimensionality reductions of the proxies of water transport capacity, morphological traits, anatomical structures, and cellular water relations. The operational rule of PCA was that all *P*-values from Bartlett’s test of sphericity were<0.001, and the extracted eigenvalues were >1. The KMO indexes ≥0.6 indicated a high reliability of the PCA result. The bivariate linear or nonlinear fitting was used to test the relationship among different principal components to reflect the compensation of the changes in morphological traits, anatomical structures, and cellular water relations to the water transport capacity. *R* ^2^ and *P*-value were used to judge the quality of the fitting mode and results. The higher the *R* ^2^ and the smaller the *P*-value (below 0.05), the better fitting result. A Pearson correlation matrix was used to analyze the relationship between all traits. All statistical analyses were conducted by R 3.4.2 software (R Core Team, 2020).

## Results

### Differences in morphological traits, anatomical structures, and cellular water relations among the three types of heteromorphic leaves

**Figure 2:**
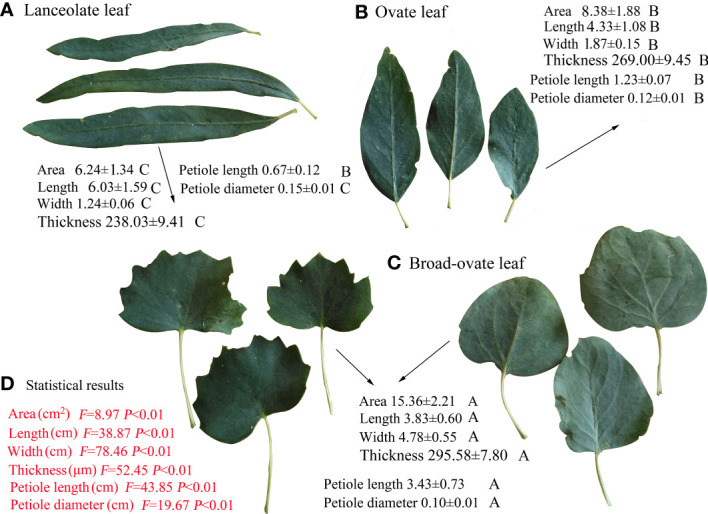
Differences in morphological traits among the three types of heteromorphic leaves. The values of *P* and *F* are the statistical results of one-way ANOVA. Different letters on the back of the related variables indicate the significant differences among heteromorphic leaves, while the same letters show the insignificant difference. Values are represented as mean ± SD.

**Figure 3:**
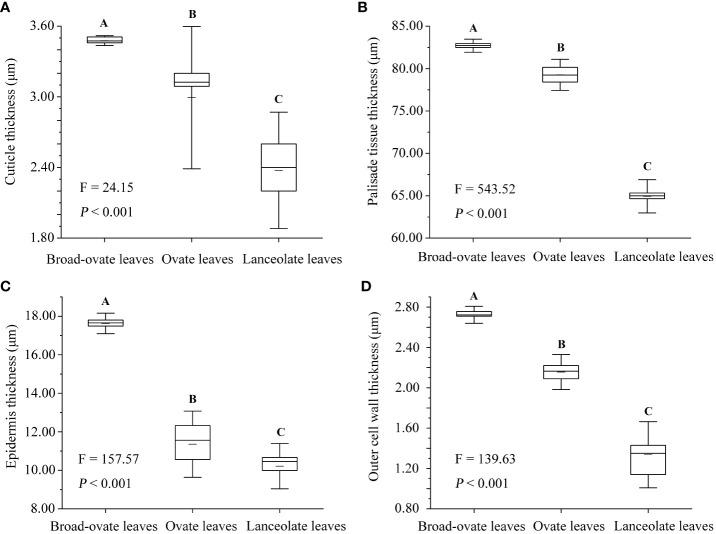
Differences in anatomical structures among the three types of heteromorphic leaves. The instructions of *P*, *F*, and other capital letters are shown in **Figure 2**.

The leaf area, leaf width, leaf thickness, and petiole length increased significantly from lanceolate to ovate and broad-ovate leaves (*P*< 0.05), whereas leaf length and petiole diameter showed an opposite pattern (*P*< 0.05) (**Figure 2**). All anatomical traits, including the thickness of the epidermis, cuticle, palisade, and outer cell wall, were all increased significantly from lanceolate to ovate and broad-ovate leaves (*P*< 0.01) (**Figure 3**).

**Figure 4:**
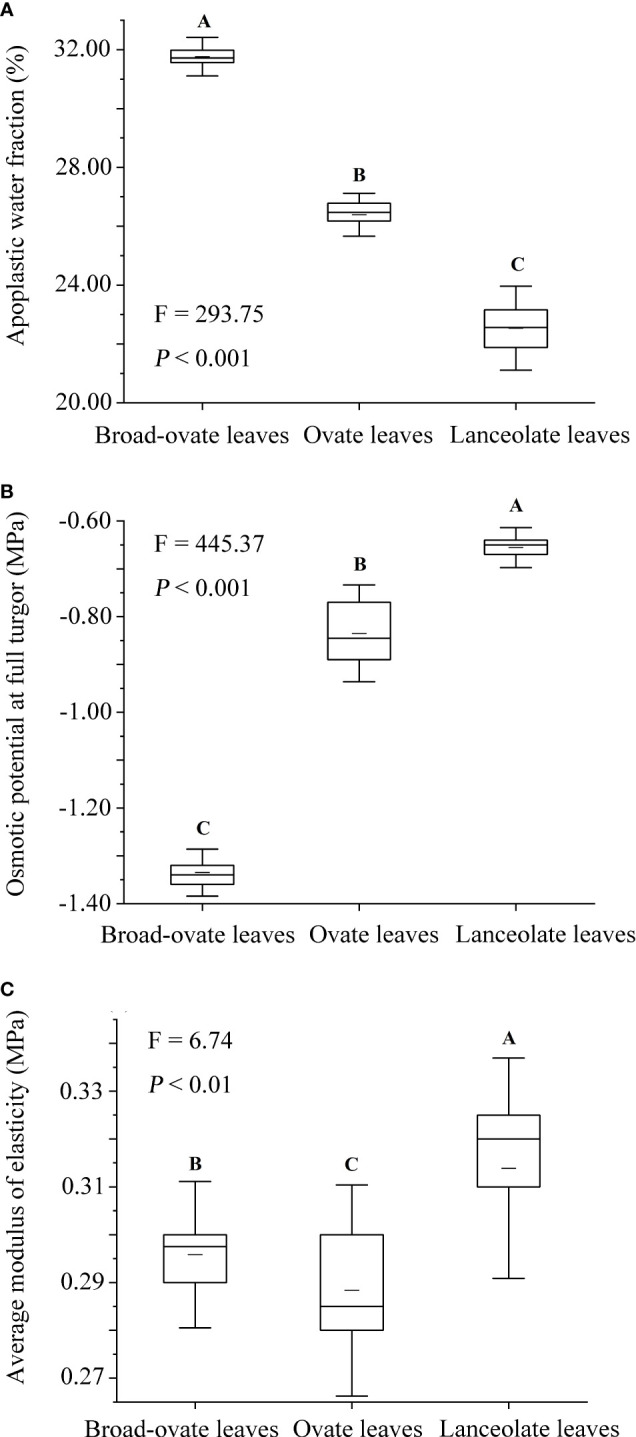
Differences in cellular water relations among the three types of heteromorphic leaves. The instructions of *P*, *F*, and other capital letters are shown in **Figure 2**.

In terms of cellular water relations, the osmotic potential at full turgor, average modulus of elasticity decreased significantly from lanceolate to ovate to broad-ovate leaves (*P*< 0.05), while the apoplastic water fraction increased (*P*< 0.05) (**Figure 4**).

### Differences in water transport capacity among the three types of heteromorphic leaves

**Figure 5:**
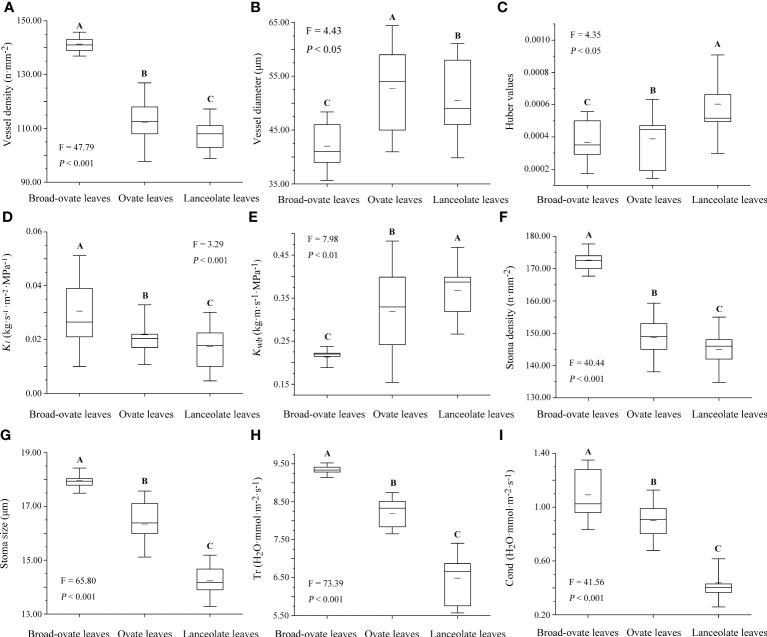
Differences in water transport capacity among the three types of heteromorphic leaves. The instructions of *P*, *F*, and other capital letters are shown in **Figure 2**.

Stem vessel density, *K_l_*, stoma size, stomatal density, Cond, and Tr were increased significantly from lanceolate to ovate to broad-ovate leaves (*P*< 0.05), while vessel diameter, *K_wb_*, and Huber values showed an opposite pattern (*P*< 0.05) (**Figure 5**).

### Relationships of water transport capacity with morphological traits, anatomical structures, and cellular water relations

**Figure 6:**
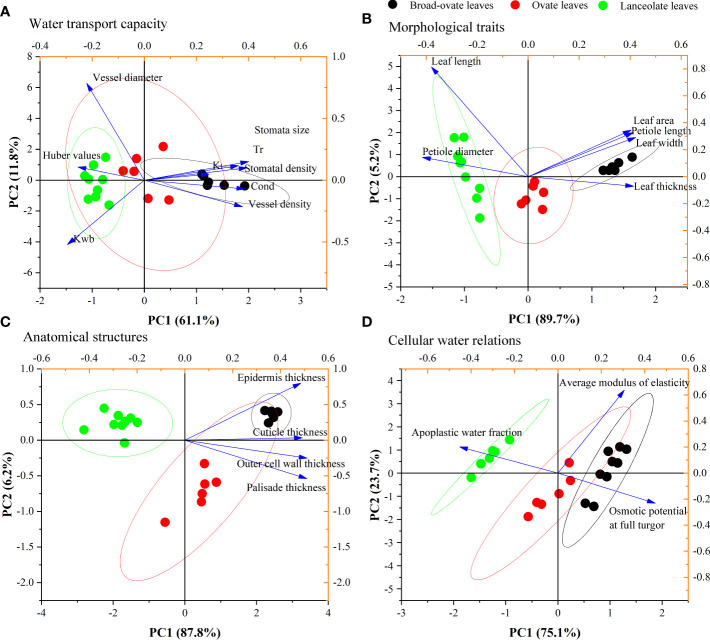
PCA results of water transport capacity, morphological traits, anatomical structures, and cellular water relations.

PCA results showed that the KMO index of water transport capacity was 0.71. Eight proxies for water transport capacity can be integrated into two principal components (PCs). PC1 and PC2 explained 61.10% and 11.80% of the total variance, respectively, with a total cumulative explanation that reached 72.90%. PC1 mainly consisted of *K_l_*, *K_wb_*, stoma size, stoma density, Cond, Tr, and vessel density, while PC2 represents vessel diameter and *K_wb_* (**Figure 6A**). PC1 and PC2 can be summed up as a trade-off between physiological water requirements (left) and the maximum water transport potential (right), as well as between hydraulic safety (up) and efficiency (down), respectively.

Similarly, PCA was used to reduce the dimension of morphological traits, anatomical structures, and cellular water relations. Our results showed that the KMO indexes of morphological traits, anatomical structures, and cellular water relations were 0.80, 0.85, and 0.57, respectively. These three aspects all can be integrated into one PC (PC1) (**Figures 6B–D**, respectively). PC1 of these three categories explained 89.75%, 87.80%, and 75.10% of their total variance (**Figures 6B–D**, respectively).

**Figure 7:**
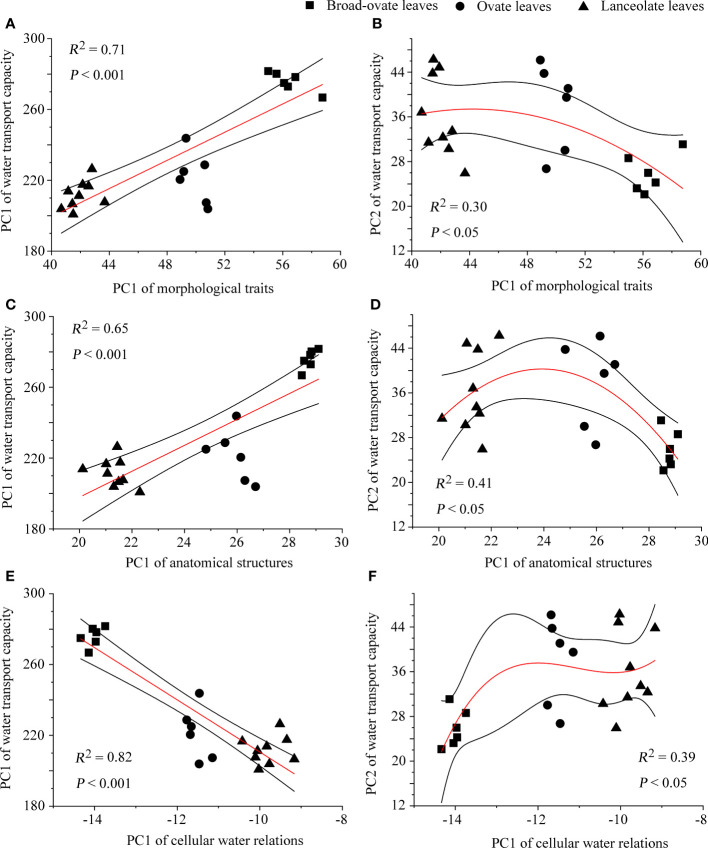
The fitting of water transport capacity with morphological traits, anatomical structures, and cellular water relations. The relationships of PC1 of water transport capacity with the other categories are performed using linear fitting, while those of PC2 are performed using exponential fitting.

The correlation matrix result showed that most of the proxies of morphological traits, anatomical structures, and cellular water relations have significantly correlated with water transport capacity (**Supplementary Figure S1**) . The fitting analysis results showed that PC1 of morphological traits, anatomical structures, and cellular water relations has a significant relationship with the two PCs (PC1 and PC2) of water transport capacity (*P*< 0.05) (**Figure 7**).

### Difference in drought stress among the heteromorphic leaves

**Figure 8:**
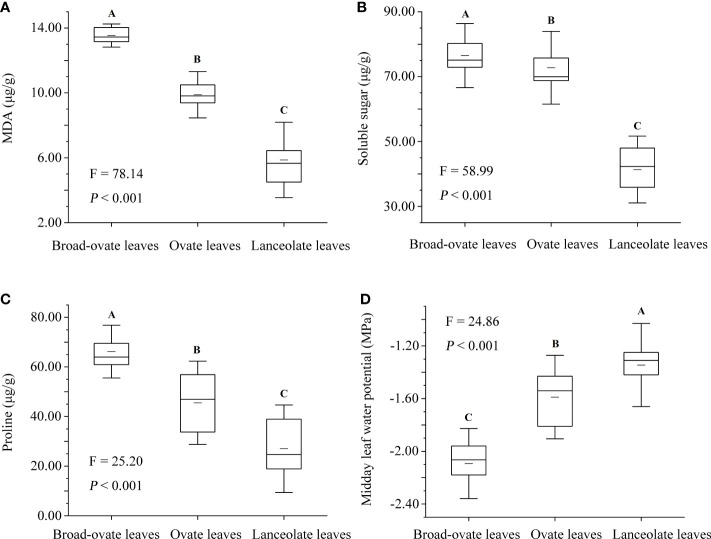
Differences in drought stress among the heteromorphic leaves. MDA is the abbreviation of malondialdehyde. The instructions of *P*, *F*, and other capital letters are shown in **Figure 2**.

The contents of MDA, proline, and soluble sugar were increased significantly from lanceolate to ovate to broad-ovate leaves (*P*< 0.01), while midday leaf water potential showed an opposite pattern (*P*< 0.01) (**Figure 8**).

## Discussion

### Variations of morphological traits, anatomical structures, and cellular water relations alleviate hydraulic constraints

Our results found that vertical distribution across height differed among the three types of heteromorphic leaves. Hydraulic constraints may affect the formation of *P. euphratica’s* heteromorphic leaves. In terms of morphological trait, *P. euphratica* tended to increase leaf area, leaf width, leaf thickness, and petiole length, while it tended to decrease leaf length and petiole diameter to alleviate the hydraulic constraints along vertical heights. Higher leaf area contained more stomata and hence increased gas exchange between the leaves and atmosphere, which finally improved water transport capacity due to the improvement of transpiration pull (Clearwater and Meinzer, 2001; Liu et al., 2019). The increase of leaf thickness while decreasing the blade aspect ratio was advantageous in transpiration water losses. This may be due to the fact that the changes in these two leaf traits would prolong the water transport distance from the main leaf vein to the abaxial mesophyll cell, thus reducing the water transport efficiency within leaf when transpiration occurs (Krt et al., 2005; Yang et al., 2022b). The increase in petiole length was crucial to adjust leaf angle, which can subsequently protect leaves from radiation injury and reduce transpiration water losses caused by direct sunlight (Niinemets et al., 2004). Based on the Hagen–Poiseuille theory, a reduction in petiole diameter would be helpful to decrease the water translocation rate and then to limit water losses due to a positive relationship between the cross-sectional area and water translocation rate (Hacke et al., 2001; Sperry et al., 2006).

Anatomical structures also have been used by *P. euphratica* to weaken hydraulic constraints. The increasing thicknesses of the palisade, cuticle, and the outer cell wall from lanceolate to ovate to broad-ovate leaves were useful in maintaining the transpiration process at a higher leaf-to-air VPD while reducing water loss synchronously (Zhuang et al., 2011; Liu et al., 2015). The increase of palisade tissue thickness changed the transfer resistance of CO_2_ from the substomatic cavity to the carboxylation site, thus maintaining photosynthesis under a high vapor pressure deficit and improving the water utilization rate (Hao et al., 2017). The large vacuoles in palisade tissue were the important water storage organ in plants (Saadeddin and Doddema, 1986). Adequate water storage in the large vacuoles can weaken the negative influence of higher vapor pressure deficit on stomatal regulation, keep stomata in open state, and continuously produce transpiration pull to maintain water transport power. As the cuticle was mainly composed of cellulose, pectin, waxes, and other polysaccharides, the increase of cuticle thickness not only prevented water loss by hindering the diffusion of water molecules from leaves to atmosphere due to the increase of filter layer but also absorbed and stuck to water molecules *via* these macromolecular components in enlarging the difficulty of water diffusion from leaf surface to atmosphere (Bacelar et al., 2004; Zhai et al., 2020). Due to a positive contribution to leaf mechanical properties, an improvement in the outer cell wall thickness protected the mesophyll cells from damage caused by rapid water loss under higher vapor pressure deficit and hence alleviated hydraulic constraints (Zhuang et al., 2011; Hao et al., 2017).

The changes in cellular water relations among the three types of heteromorphic leaves also contributed to alleviating hydraulic constraints. Apoplastic water acted as a buffer for cell water during dehydration. A high apoplastic water fraction suggested that mesophyll cells can tolerate higher water deficits by storing more water (Gunasekera and Berkowitz, 1992; Yang et al., 2005). The osmotic potential at full turgor was closely related to the capacity of osmotic adjustment. Lower osmotic potential at full turgor indicated that mesophyll cells have a wide range of osmoregulation, ensuring that the higher vapor pressure deficit had not caused the cells to lose too much water to recover (Huo et al., 2021). A mesophyll cell with a higher average modulus of elasticity might be more effective in maintaining structural integrity during rehydration after a period of high vapor pressure deficit (Chartzoulakis et al., 2002; Sassi et al., 2013). The increase of the average modulus of elasticity was conducive to increase the expansion capacity of cells, hence maintaining turgor pressure and alleviating the damages of higher vapor pressure deficit on cellular organelles (Sassi et al., 2013; Huo et al., 2021).

Current studies suggested that plants mainly used some adaptive strategies to alleviate the increasing hydraulic constraints along the vertical height (Bucci et al., 2004; Ryan et al., 2006; Preisler et al., 2021). The well-known theory was that plants usually closed some stomata on the leaves at the top of the crown to reduce the water exchange between leaves and atmosphere to relieve the negative pressure in the xylem vessel, thus weakening hydraulic constraints (Koch et al., 2004; Yang et al., 2019c). However, water transport capacity also decreased in this process due to stomatal closure and the reduction of transpiration pull (Ryan and Yoder, 1997; Manzoni et al., 2013). The secondly well-known view was that when the negative pressure in the xylem vessel has exceeded the threshold of embolism formation, plants will not close the stomata on the leaves at the top of the crown (Mcdowell et al., 2010). The opposite was to continue to increase transpiration during the day and retain the excess water in the water storage tissue (Ryan et al., 2006; Ishii et al., 2014). Although the risk of embolism was increased during this process, the stored water subsequently was likely to form a positive pressure in the xylem vessel at night when transpiration was weak, which subsequently repaired the embolism (Bucci et al., 2004; Preisler et al., 2021). Unlike the first adaptation strategy, transpiration pull had not decreased during this process because stomata were not closed. Instead, plants may use stored water to keep stomata open and increase transpiration pull to overcome the negative effect of hydraulic constraints on water transport from stem to leaves, or in stems (Ishii et al., 2014; Himeno et al., 2017). The upper broad-ovate leaves have better cellular water relations and thicker outer cell wall and palisade tissue compared with the other leaf types, indicating that their mesophyll cells can store more water and suffer higher vapor pressure deficit. These demonstrated that *P. euphratica* may adopt the strategy of water storage to alleviate the increasing hydraulic constraints along the vertical height.

The water storage strategy was used to reduce hydraulic constraints of *P. euphratica* that can also be proven by the variations of stomatal size and density, Tr, Cond, and Huber value among the three types of heteromorphic leaves. As the height increased, *P. euphratica* had not closed the stomata at the top of the crown to reduce hydraulic constraints. Oppositely, it may use stored water to realize rapid transpiration rates. This conclusion was consistent with many studies on water storage species (Bucci et al., 2004; Himeno et al., 2017). In terms of species with water storage capacity, although the efficiency of water transfer from stem to leaves was reduced, such as the decrease of Huber value and leaf specific conductance (*K_l_*), the use of water storage can ensure that the leaves at the top of the crown tolerated higher vapor pressure deficit. The broad-ovate leaves of *P. euphratica* owned higher stomatal size and density than ovate and lanceolate leaves. Driven by leaf-to-air VPD, the advantage of stomatal size and density made the upper leaves possess higher transpiration rate and stomatal conductance compared with the lower leaves, resulting in greater transpiration pull and water transport capacity, which lifted more water from soil to the top of the crown (Hao et al., 2017; Amitrano et al., 2021). Another pivotal reason for the upper leaves to maintain higher stomatal size and density may be to improve latent cooling *via* transpiration and avoid leaf overheating because they suffered from higher heat stress caused by more direct sunlight (Urs and Vaseva, 2014). But this strategy was different from the hypothesis of Franks and Beerling (2009). They considered that there should be a trade-off between stomatal density and size, since there was a theoretical upper limit to stomatal size at a given density, or an upper limit to density at a given size. The possible reason why *P. euphratica* had not fitted the hypothesis was that it owned great phenotypic plasticity. The morphology, physiology, and anatomical structure were quite different between the heteromorphic leaves. Such enormous phenotypic plasticity may make homogeneity less limiting to leaf trait variation (Li et al., 2017), resulting in the trade-offs of paired leaf traits, such as stomatal size and density, that do not exhibit obvious coevolutionary relationships.

Changes in leaf morphology, cellular water relations, and anatomical structures also can improve water transport capacity *via* affecting the transpiration and hydrodynamic processes in leaves (Bacelar et al., 2004; Krt et al., 2005; Garcia et al., 2021). These can be confirmed by our PCA results. Our study found that nine traits related to water transport can be grouped into two aspects: a trade-off between physiological water requirements and the maximum water transport potential (PC1) and the other between hydraulic safety and efficiency (PC2). These two aspects all significantly correlated with leaf morphology, cellular water relations, and anatomical structures. This was similar to the research results of Song et al. (2022) and Yang et al. (2022a). The actual water transport capacity of plants was determined by both environmental stress and maximum water transport potential. With the increase of environmental stress, especially drought stress, plants can adjust trade-off between hydraulic safety and efficiency by changing the leaf functional traits to increase the possibility of survival. Moreover, inconsistent change patterns between *K_l_* and *K_wb_* in PCA (**Figure 6A**) also confirmed that plants can adjust their water transport capacity by changing the leaf shape, leaf physiological traits, and anatomical structure. *K_l_* was not only related to the instantaneous water conductivity (*K_wb_*) and the maximum stem-specific hydraulic conductivity but also related to the total area of leaves growing on the branch and the anatomical structure and physiological characteristics of the leaves. For example, even though *K_wb_* and *K_l_* are the same, the larger total area of broad-ovate leaves on the branch will make the *K_l_* value associated with it smaller than the other two kinds of heteromorphic leaves.

### The manipulation of hydraulic constraints on heteromorphic leaf was facilitated by drought stress

All woody plants in arid deserts, especially arborescent species, would suffer hydraulic constraints with the increase of height (Yang et al., 2019a; Wang et al., 2021). Variations in leaf area, blade aspect ratio, anatomical structure, and cellular water relations have also been observed in many plants in response to hydraulic constraints (Bucci et al., 2004; Leigh et al., 2011; Males, 2017), but heteromorphic leaves rarely appeared in the tree species in the terrestrial ecosystems, for example, changed leaf shape from lanceolate to ovate. Different types of heteromorphic leaves grow at different vertical heights of *P. euphratica*, which may be related to the great drought stress on the arid desert ecosystem (Liu et al., 2015; Iqbal et al., 2017; Zhai et al., 2020). *P. euphratica* was the only natural tree species in the arid desert community, and its potential height was larger than that of other species (Yang et al., 2014b). *P. euphratica* may be the species suffering the highest hydraulic constraints in the arid desert community. In addition, *P. euphratica* has been subjected to long-term drought stress due to the serious shortage of local precipitation (Hao et al., 2017; Keram et al., 2019). With the increase of tree height, water supply of upper leaves was more difficult than that of lower leaves, resulting in more drought stress. For example, several indicators for assessing the degree to which plants are subjected to drought stress (i.e., the midday leaf water potential and the contents of MDA, proline, and soluble sugar) of the broad-ovate leaves all differed from those of ovate and lanceolate leaves (**Figure 8**). Drought stress will reduce leaf water potential and aggravate water vapor pressure deficit, further aggravating hydraulic constraints (Keram et al., 2019; Li et al., 2019). In this scenario, *P. euphratica* might adopt extreme leaf shape variations (heteromorphic leaves) to reduce the increase of hydraulic constraints along vertical heights.

## Conclusions and prospects

This study demonstrates that heteromorphic leaves of *P. euphratica* were distributed differentially along vertical heights. Water transport capacity was higher in broad-ovate leaves than that in lanceolate to ovate leaves. The changes of morphological traits, anatomical structure, and cellular water relations from lanceolate to ovate to broad-ovate leaves were conducive to reducing transpiration water loss and increasing hydraulic efficiency. Additionally, water transport capacity was obviously related to morphological traits, anatomical structure, and cellular water relations. This study concludes that the changes in the spatial distribution of heteromorphic leaves might be determined by hydraulic constraints in the arid desert region. This study provides new insight into the formation of heteromorphic leaves and a theoretical basis for desert plants to adapt to drought stress. However, our study also had some shortages. For example, wind disturbance and radiation stress may also play a role in the vertical distribution of heteromorphic leaves. This is due to the fact that the change of leaf morphology is beneficial to increase light interception and reduce windage area. However, relationships between wind disturbance, radiation stress, drought stress, hydraulic constraints, and spatial distribution of heteromorphic leaves were not included totally in our study. More work needs to be done in revealing the formation of *P. euphratica*’*s* heteromorphic leaves in the future.

## Data availability statement

The raw data supporting the conclusions of this article will be made available by the authors, without undue reservation.

## Author contributions

All authors designed the study. EA, X-DY, and JZ collected the data. EA, AA, Y-LX, L-BS, X-WG, Y-CG, YL, and PG quantified recordings, ran statistical analyses and drafted the manuscript. All authors read, revised and approved the manuscript

## Funding

This work was supported by the National Natural Science Foundation of China (Grant Nos. 31860111 and 41871031), and Ningbo Natural Science Foundation (Grant No. 202003N4133).

## Acknowledgments

The authors thank Yan-Xin Long, Ya-Yun Wang, Heng-Fang Wang, La-Mei Jiang, Qi Yang and the members of Key Laboratory of Oasis Ecology of Xinjiang University for their indispensable help in fieldwork and laboratory analysis.

## Conflict of interest

The authors declare that the research was conducted in the absence of any commercial or financial relationships that could be construed as a potential conflict of interest.

## Publisher’s note

All claims expressed in this article are solely those of the authors and do not necessarily represent those of their affiliated organizations, or those of the publisher, the editors and the reviewers. Any product that may be evaluated in this article, or claim that may be made by its manufacturer, is not guaranteed or endorsed by the publisher.
