# Supplementary material for: Hydraulic constraints determine the distribution of heteromorphic leaves along plant vertical height
Source: Front Plant Sci. 2022 Sep 29;13:941764. doi: 10.3389/fpls.2022.941764 (PMC9580785; doi:10.3389/fpls.2022.941764)

**Hydraulic constraints determine the distribution of heteromorphic leaves along plant vertical height**

**Xiao-Dong Yang^1,2^, Elhamjan Anwar^2^, Yi-Lu Xu^3^, Jie Zhou^2^, Long-Bin Sha^2^, Xue-Wei Gong^4^, Ali Arshad^5^, Yong-Chao Gao^6^, Yanju Liu^3^ and Ping Ge^7^***

^1^Department of Geography and Spatial Information/Center for Land and Marine Spatial Utilization and Governance Research, Ningbo University, Ningbo, China, ^2^Institute of Resources and Environment Science, Xinjiang University, Urumqi, China,

^3^Global Centre for Environmental Remediation (GCER), The University of Newcastle (UON), Newcastle, NSW, Australia,

^4^Institute of Applied Ecology, Chinese Academy of Sciences, Shenyang, China, ^5^Forest Ecology Research Group, College of Life Sciences, Hebei University, Baoding, Hebei, China,

^6^Shandong Provincial Key Laboratory of Applied Microbiology, Ecology Institute, Qilu University of Technology (Shandong Academy of Sciences), Jinan, China, ^7^Department of Development Planning, Zhejiang Gongshang University, Hangzhou, China

***Corresponding author:** **Ping Ge**. [geping2010@126.com](mailto:geping2010@126.com).

**Running title:** Heteromorphic leaves

**Figure S1.** Pearson Correlation among water transport capacity, morphological traits, anatomical structures and cellular water relations of heteromorphic leaves. Stoma density (SD) and size (SS), stem xylem anatomy (vessel diameter and density/VDIA and VDEN), Huber value (HV), the instantaneous water conductivity (*K_wb_*) of branches, the leaf specific hydraulic conductance (*K_l_*), and transpiration traits (transpiration rate and stomatal conductance/ Tr and Cond) were used as proxies for water transport capacity. Morphological traits included the area (LA), thickness (LT), length (LL) and width (LW) of leaf, and petiole length (PL) and diameter (PD). Anatomical structures were composed of the thickness of epidermis (ET), cuticle (CT), palisade (PT), and outer cell wall (OCWT). Cellular water relations included the apoplastic water fraction (AWF), osmotic potential at full turgor (OPFT), and average modulus of elasticity (AME).


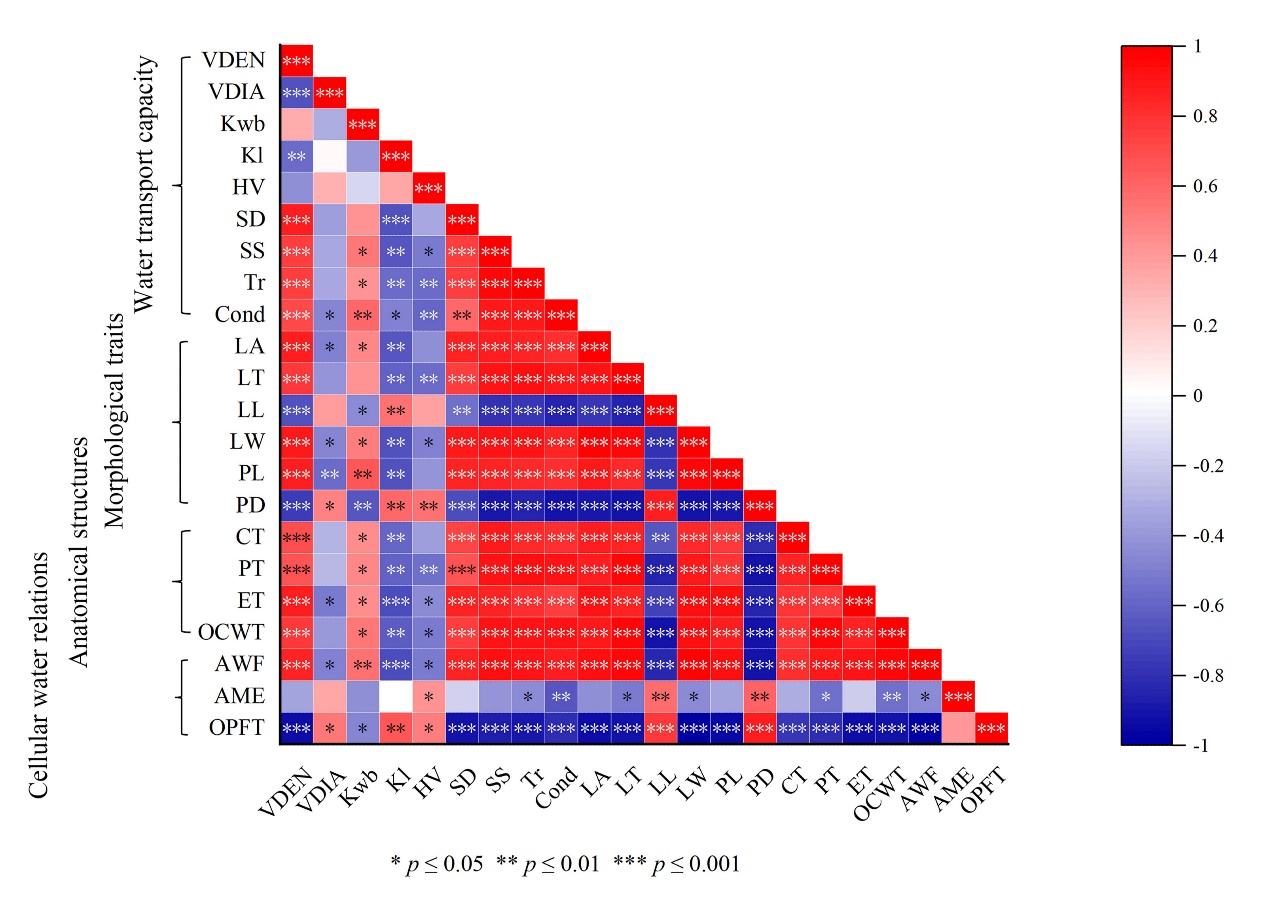

Supplement: Supplementary file 1 [file DataSheet_1.docx]
